# Contact Heterogeneity, Rather Than Transmission Efficiency, Limits the Emergence and Spread of Canine Influenza Virus

**DOI:** 10.1371/journal.ppat.1004455

**Published:** 2014-10-23

**Authors:** Benjamin D. Dalziel, Kai Huang, Jemma L. Geoghegan, Nimalan Arinaminpathy, Edward J. Dubovi, Bryan T. Grenfell, Stephen P. Ellner, Edward C. Holmes, Colin R. Parrish

**Affiliations:** 1 Department of Ecology and Evolutionary Biology, Princeton University, Princeton, New Jersey, United States of America; 2 Baker Institute for Animal Health, College of Veterinary Medicine, Cornell University, Ithaca, New York, United States of America; 3 Marie Bashir Institute for Infectious Diseases and Biosecurity, Charles Perkins Centre, School of Biological Sciences and Sydney Medical School, University of Sydney, Sydney, New South Wales, Australia; 4 Department of Ecology and Evolutionary Biology, Princeton University, Princeton, New Jersey, United States of America; 5 Department of Infectious Disease Epidemiology, School of Public Health, Imperial College London, South Kensington Campus, London, United Kingdom; 6 Department of Population Medicine and Diagnostic Sciences, College of Veterinary Medicine, Cornell University, Ithaca, New York, United States of America; 7 Fogarty International Center, National Institutes of Health, Bethesda, Maryland, United States of America; Centers for Disease Control and Prevention, United States of America

## Abstract

Host-range shifts in influenza virus are a major risk factor for pandemics. A key question in the study of emerging zoonoses is how the evolution of transmission efficiency interacts with heterogeneity in contact patterns in the new host species, as this interplay influences disease dynamics and prospects for control. Here we use a synergistic mixture of models and data to tease apart the evolutionary and demographic processes controlling a host-range shift in equine H3N8-derived canine influenza virus (CIV). CIV has experienced 15 years of continuous transfer among dogs in the United States, but maintains a patchy distribution, characterized by sporadic short-lived outbreaks coupled with endemic hotspots in large animal shelters. We show that CIV has a high reproductive potential in these facilities (mean R_0_ = 3.9) and that these hotspots act as refugia from the sparsely connected majority of the dog population. Intriguingly, CIV has evolved a transmission efficiency that closely matches the minimum required to persist in these refugia, leaving it poised on the extinction/invasion threshold of the host contact network. Corresponding phylogenetic analyses show strong geographic clustering in three US regions, and that the effective reproductive number of the virus (R_e_) in the general dog population is close to 1.0. Our results highlight the critical role of host contact structure in CIV dynamics, and show how host contact networks could shape the evolution of pathogen transmission efficiency. Importantly, efficient control measures could eradicate the virus, in turn minimizing the risk of future sustained transmission among companion dogs that could represent a potential new axis to the human-animal interface for influenza.

## Author Summary

Influenza virus infects a range of vertebrate hosts, including domesticated animals as well as humans. Some of the most serious influenza pandemics in humans have involved host range shifts, when an influenza virus jumps from one host species to another. Importantly, however, host range shifts do not always cause pandemics. Rather, epidemiological patterns tend to be unpredictable in new host species, causing disease patterns that change over space and time. In this paper, we analyze epidemiological and evolutionary dynamics of canine influenza virus (CIV), which jumped to dogs in the late 1990s from an equine strain (EIV) prevalent in horses. We show that the epidemiology and evolution of CIV is strongly influenced by heterogeneous patterns of infectious contact among dogs in the US. A few large populations in metropolitan animal shelters serve as reservoirs for CIV, but the virus cannot be maintained for long in smaller facilities or in the companion dog population without input from the larger shelters, which represent disease hotspots. These hotspot dynamics give a clear picture of what can happen in the time between the beginning of a host range shift and the onset of a possible pandemic, allowing more targeted strategies for control and eradication.

## Introduction

Respiratory pathogens that emerge as the result of host-range shifts can cause serious epidemics in humans, livestock, and wild animals [1]–[4]. Two recent pandemics in humans – Severe Acute Respiratory Syndrome (SARS) in 2003 and H1N1 influenza in 2009 – involved host-range shifts in respiratory zoonotic viruses [5], [6], while the recently documented Middle East respiratory syndrome coronavirus (MERS-CoV) has similarly emerged from an animal reservoir to pose a growing risk to the human population [7]. Importantly, however, cross-species transmission events do not always lead to pandemics. Rather, zoonoses emerging in new host species tend to have patchy and dynamic prevalence patterns in space and time. As a result, the probability that an emerging zoonosis will take hold in a new host population has been difficult to assess *a priori*, which limits our capacity to use targeted interventions to avert pandemics before they happen [8].

A variety of host-pathogen interactions may follow a species jump, and revealing their determinants is essential to understanding the process of zoonotic emergence. First, the emerging pathogen may be poorly adapted for replication and onward transmission in the new host population. This leads to inefficient transmission, where many potentially infectious contacts between susceptible and infected individuals fail to spread the disease, due for example to a low pathogen load in the infected individual. In this case, the disease will have a lower basic reproductive number (R_0_ – the number of secondary infections caused by a typical infected individual in an entirely susceptible population) in the recipient host than its recent ancestor in the donor host. Inefficient transmission following a spillover event may lead to “stuttering chains” of infection marked by patchy patterns of disease prevalence interspersed with stochastic fadeouts. Even if a pathogen has R_0_ above 1 (a necessary but not sufficient condition for self-sustaining spread), values of R_0_ that are only marginally above 1 are associated with a higher probability of stochastic extinction. The probability that a pathogen will establish itself (in a large homogeneously mixed population of susceptible hosts) following the introduction of *n* infected individuals is given by 1−(1/R_0_)*^n^* [9].

The heterogeneity in prevalence of emerging pathogens may also reflect the demographic variability inherent to host populations. In smaller host populations random variation in the timing and frequency of births, deaths, immigration, emigration, and contacts between infected and non-infected individuals, as well as in the timing of individual infections, can have profound effects on epidemic dynamics [10]–[12]. Emerging pathogens that result from spillover into new hosts are by definition initially confined to a small population, in the sense that the first infected individual(s) will have limited numbers of potential contacts to whom they can spread the disease. This, in turn, makes the epidemic dynamics of emerging pathogens inherently stochastic [13], [14].

Finally, evolutionary change in emerging pathogens can affect both their basic reproductive number, and their response to demographic variability. Pathogen evolution can result in R_0_ increasing toward or above 1.0 after repeated spillover events from the reservoir population, or during a chain of transmission in the new host, either of which could result in the emergence and selection of host-adaptive mutations. The occurrence of multiple outbreaks over time may also increase the likelihood that the pathogen evolves toward a point when it can be self-sustaining in the new host [12]. Recent analytical frameworks that unite the ecological and evolutionary dynamics of host-pathogen interactions can help identify the processes that drive epidemiological and phylogenetic patterns during and after host-range shifts [11], [12], [15].

Here we study the population dynamics and evolution of equine-H3N8 derived canine influenza virus (CIV) in the US, and use the results to propose control strategies. CIV emerged following the transfer of a single H3N8 equine influenza (EIV) to dogs from horses around 1999. Direct descendants of that virus have been circulating continuously in dogs since that time [16]–[18]. CIV was first recognized as the cause of disease in greyhounds in a training facility in Florida in 2004 and was transferred to various states in the US with the racing greyhounds, eventually spreading to other breeds [16]. The hemagglutinin (HA) sequence of CIV was genetically distinct from EIV by 2004, forming a separate monophyletic group from EIV in phylogenetic trees [16]. Notably, there is no evidence of CIV transfer back to horses, onward to humans, nor of reassortment involving CIV and other influenza viruses [19]. Furthermore, although some other H3N8 EIV spillovers from horses into dogs have been reported, these only comprised single infections or small outbreaks that rapidly faded out [20].

Although CIV can readily transmit among dogs its prevalence remains patchy: it is enzootic in some regions of the US, but has thus far failed to establish outside of these enzootic regions [21]–[24]. The overall seroprevalence of CIV in the companion (pet) dog population appears to be low (∼3% or less depending on the region), with visits to canine daycare a risk factor [22], [24]. CIV enzootic regions are typically associated with large animal shelters [25], and the movement of the virus to different parts of the US is most likely associated with the transport of infected shelter dogs to facilities in other regions where they may be more readily adopted [26].

In contrast to CIV, its recent ancestor, H3N8 EIV, has been circulating widely in horses since before 1963 when it was first reported in Florida, having most likely been introduced with horses from South America [27]. The virus appears to spread continuously within and between many parts of North and South America, Europe, and Asia [28]–[31]. EIV has been introduced into countries that were previously free of the virus, including Australia and South Africa, causing significant outbreaks that extended over large distances, although these were controlled and the virus eradicated [28], [32]. Data from an outbreak in an unvaccinated population of racehorses places R_0_ for EIV at 10.18 (95% confidence interval: 9.57–10.89) in that context. In contrast, the reproductive number of EIV in vaccinated populations of racehorses has been estimated to be between 1.4 and 2.3 [33]. EIV has experienced marked evolution in all gene segments since it emerged, with evidence of antigenic variation in the HA gene, including phylogeographic patterns in HA variation, with distinct clades in Europe versus the US, and among US states [28], [34], [35].

Although CIV and EIV are closely related, their epidemiology and evolutionary dynamics differ, with EIV seemingly more successful, and less heterogeneously distributed. Moreover, EIV continues to spread despite active control measures (particularly vaccination) whereas CIV retains a patchy distribution in the absence of significant control measures. An analysis of the phylogenetic history and ecology of CIV since its recent emergence from EIV may therefore reveal how host demography, disease dynamics, and pathogen evolution can combine to determine the prevalence patterns and risk posed by emerging zoonotic pathogens.

Here we combine individual-level data on the intake, output, and transfer rates of dogs among US animal shelters of different sizes, with CIV gene sequence data and available seroprevalence estimates, to identify the processes controlling disease dynamics in emerging zoonoses at the human-animal interface. We hypothesize that CIV persists through the presence of transmission hotspots, which rescue chains of transmission that fade out in other populations. The putative hotspots are large animal shelters in major metropolitan areas. After estimating R_0_ from all available data we ask: are the population sizes of small shelters small enough to make fadeout significantly more likely than in large shelters? And do large shelters have good prospects of maintaining CIV in an enzootic state?

We then use the parameters from our analyses to determine what control measures would result in eradication of the virus. CIV is a prime target for eradication because it has both the potential to cause significant disease burden, and it is currently confined to a small subset of its host population. Effectively controlling CIV would improve conditions in metropolitan animal shelters, as well as minimizing the risk of zoonotic human infection posed by CIV, before it has the opportunity to evolve higher transmissibility in the companion dog population. A possible future scenario of sustained CIV transmission amongst companion animals would represent the evolution of a potentially significant new axis to the human-animal interface for influenza.

## Results

### Phylogenetic Structure of CIV in the USA

To put the CIV sampled from animal shelters in a wider geographical context, and to reveal movement of the virus on a continental scale, we determined the HA1, M and NP gene sequences of recent CIV isolates, and conducted a phylogenetic analysis of these sequences combined with homologous sequences available on GenBank as well as the Influenza Research Database. Viruses or sequences were sampled from the US states of Colorado, New York, Pennsylvania, Florida, California, Kentucky, Wyoming, Philadelphia, South Carolina, Virginia, Vermont, Connecticut, Texas, and Iowa.

**Figure 1:**
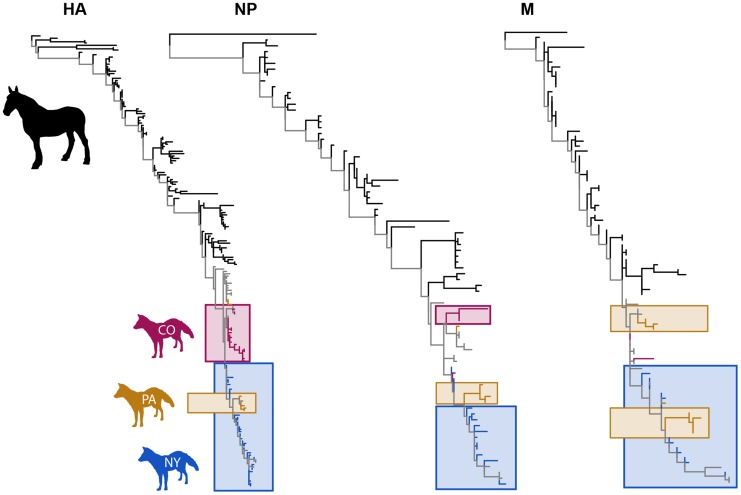
Phylogenetic trees of HA1, NP and M sequences for EIV (black) and CIV (colors). Boxes surround CIV clades comprising two or more samples from the same US state. Branches leading to CIV samples from the same location are colored by location (New York, blue; Pennsylvania, orange; Colorado, purple. Branches leading to CIV samples from multiple locations are colored grey.

The most striking result of this analysis is that CIV exhibits a marked geographical clustering by US state with distinct clades being observed in New York (and nearby states), in Pennsylvania, and in Colorado, which also represent our largest sampling sets (Figure 1). This geographical clustering was confirmed in Association Index (AI) and Parsimony Score (PS) phylogeny-trait association statistics [36], with significantly more clustering by US state of origin than expected by chance alone across the data set as a whole (p<0.001). Similarly, the Maximum Clade (MC) statistic reveals significant (p<0.001) clustering in the individual states of Colorado, New York, Pennsylvania, Vermont and Wyoming. In addition, many of the viruses from the northeastern states of Vermont, Connecticut, New Hampshire clustered with the viruses that are circulating continuously in New York (also in the northeast), suggesting that those viruses were derived from the New York enzootic hotspots.

### Population Structure and Epidemiological Dynamics of CIV in Shelter and Companion Dogs

Next, we investigated the demographic and epidemiological dynamics of CIV at the local scale in animal shelters. Here we used individual-level records of dog arrival and departure from 13 animal shelters of varying size across the US, comprising a total of 124,519 dogs, as well as published seroprevalence estimates from a large shelter [25], coupled with a stochastic epidemic model. The epidemic model was an SIR-type model incorporating empirical rates of arrival and departure from the shelter as well as CIV infection and removal dynamics, and implemented at the level of individual dogs using the Gillespie algorithm [37].

**Figure 2:**
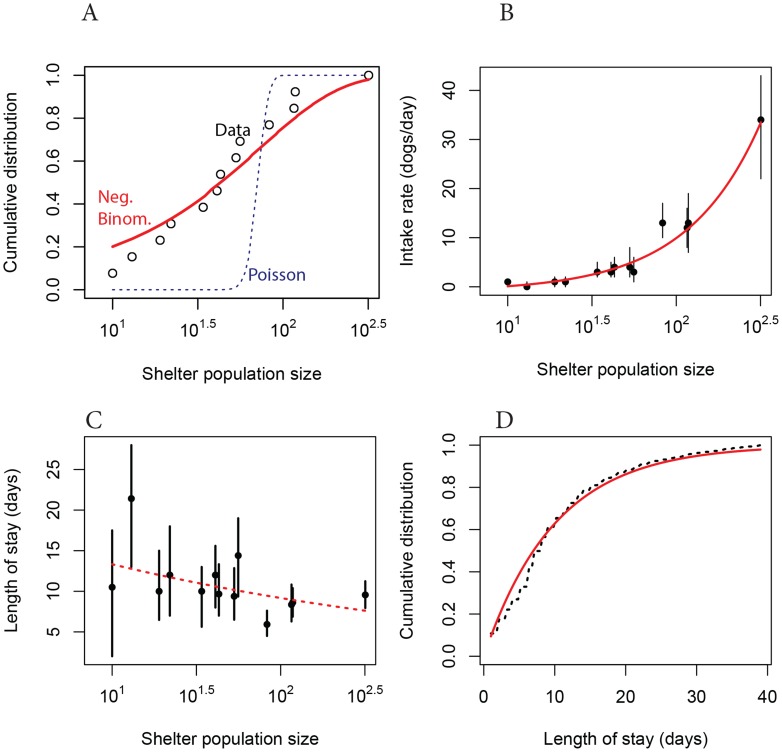
Demography of dogs in US animal shelters. (**A**) Cumulative distribution of median population size in each shelter (black dashed line) compared to a negative binomial distribution fitted to the data (solid red line), and a fitted Poisson distribution (dotted blue line). (**B**) Intake rate as a function of population size. Points show the median value for each shelter and vertical lines enclose the interquartile range. Line shows fit by linear regression to log-transformed median intake rates. (**C**) Length of stay as a function of shelter size. The 95% confidence interval on the slope of the dashed line includes 0. (**D**) Cumulative distribution of length of stay across all shelters (bars) compared to an exponential distribution with mean rate 1/9.88 days^−1^ (solid line).

The majority of animal shelters in the US house relatively small populations of dogs—the median dog population size in our sample of shelters is 43—but a few shelters are much larger, housing hundreds of dogs. In precise terms, the distribution of dog population sizes in our data is close to a negative binomial distribution with mean 71.23 and standard deviation 82.24 (Figure 2A), which indicates significant overdispersion in population sizes relative to a homogeneous Poisson model. This overdispersion in host population size is a potentially important characteristic for the epidemiology of CIV because it indicates the presence of a few extraordinarily large shelters where a pathogen might persist more easily than in a host population of average size. Large well-connected populations are more favorable environments for emerging pathogens because variance in vital rates (e.g. the rate of arrival of new susceptible individuals) decreases predictably with population size. All else being equal, this makes large host populations more stable for sustained pathogen transmission.

Shelters with larger populations are fueled primarily by higher intake rates (Figure 2B), as the median residence time of dogs does not vary significantly among shelters of different sizes (Figure 2C). The residence time of dogs in a shelter is roughly exponentially distributed with a mean of 9.88 days and a standard deviation of 8.22 days (Figure 2D). Transfer rates among shelters appear relatively low—among the eight shelters in our demographic data for which there was transfer information the median proportion of dogs whose stay at a shelter ended with a transfer is 0.067 and the mean is 0.1. Transfer probability is not correlated with dog population size in our data (data not shown).

**Figure 3:**
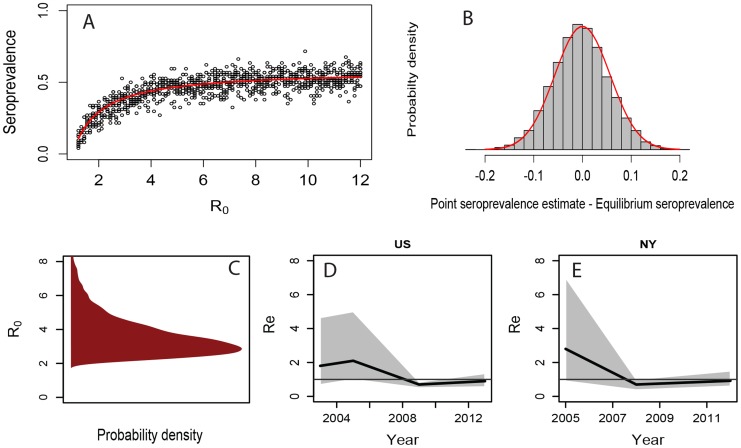
Seroprevalence, R_0_ and R_e_ for CIV, estimated from host demographic data, seroprevalence data, and molecular data. (**A**) Saturating relationship between seroprevalence and R_0_ in a stochastic SIR framework, parameterized from the shelter intake and output data. Red line shows equilibrium seroprevalence predicted by the mean-field model. Points show point seroprevalence estimates from the stochastic simulations, where 74 dogs are sampled at random in a shelter with an average dog population of 134, corresponding to [25]. (**B**) Deviations of point seroprevalence estimates from the long-term average (bars) compared to a normal distribution (line). (**C**) Posterior distribution of R_0_ based on an observed seroprevalence of 0.42 in [25]. (**D**) and (**E**) R_e_ for CIV, estimated by fitting a birth-death skyline phylodynamic model to HA1 gene sequences. The black line shows the mean estimate while the grey shaded shows the highest posterior density (HPD) range, encompassing 95% of the credible set of sampled values.

Most dogs arriving to shelters are susceptible to CIV [22], [25]. The arrival rate of susceptible dogs places an upper limit on CIV prevalence by continual dilution with uninfected individuals, which leads to a saturating relationship for prevalence as a function of R_0_ (Figure 3A). We estimated a posterior distribution for R_0_ given seroprevalence data and demographic data by using a Markov Chain Monte Carlo (MCMC) method based on a stochastic SIR model parameterized with the demographic data (see Methods). Point estimates of seroprevalence are normally-distributed about the long-term equilibrium value given by the mean-field model in our simulations (Figure 3B), and a seroprevalence estimate of 0.42 [25] from a large shelter where CIV is enzootic, combined with the demographic data on dog intake and outcome rates, yield a mean estimate for R_0_ of 3.9 for CIV in large animal shelters. The posterior distribution of R_0_ has a median of 3.3, and a highest probability density (HPD - the central 95% of the posterior distribution) interval extending from 2.0 to 8.9 (Figure 3C).

Moving from estimating the reproductive potential (R_0_) of CIV in animal shelters to estimating the effective spread rate in the general population from genetic data, we employed phylodynamic birth-death models to analyze HA1 sequence data collected across the US to determine the rate of spread of the virus in the wider dog population. Our estimates for the effective reproductive number (R_e_; the average number of secondary infections produced by a typical infected individual at a given time, in a population where not all potential hosts are necessarily susceptible) show considerable temporal variation (Figure 3D,E). At the time when CIV was first recognized in 2004 the posterior distribution of R_e_ roughly matches that of R_0_, consistent with the initial, exponential phase of the epidemic. During the period 2004–2008 R_e_ drops to a value of approximately 1.0. A similar pattern was observed in the New York data set. Across the USA as a whole the mean estimate of R_e_ is currently 1.02 (95% HPD = 0.79,1.26), with a similar number found in New York (R_e_ = 1.06, 95% HPD = 0.72, 1.47) (Figure 3). The low R_e_ observed toward the present suggests that CIV spread has now reached an equilibrium, where stochastic fadeouts often associated with outbreaks are balanced with new infections, which usually occur in the large animal shelters where it is enzootic.

### Populations That Sustain Viral Transmission

**Figure 4:**
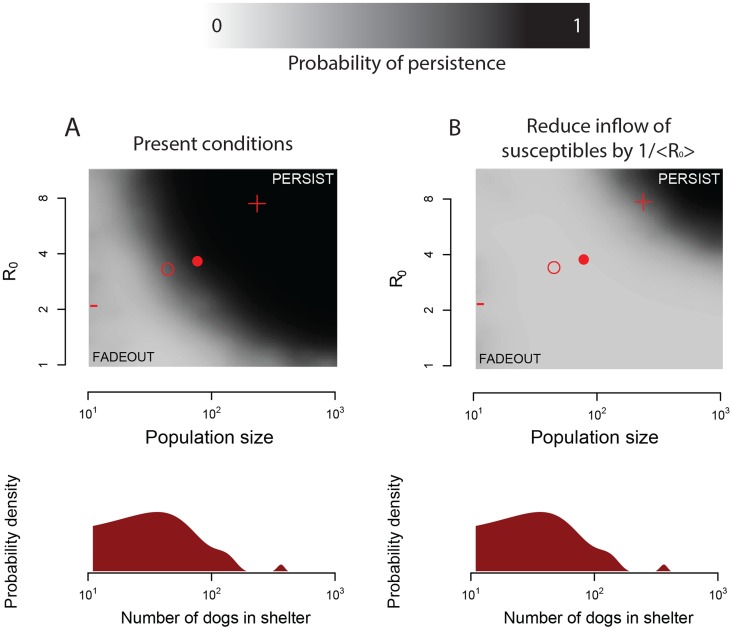
Demographics, persistence, spread rate and possible eradication of CIV. (**A**) Dog population sizes in animal shelters and within-shelter spread rates at which CIV can persist for at least 100 days according to present intake and output rates. The surface shows a smoothed (kriged) version of the outcome (persistence for at least 100 days) of 1000 simulations conducted at uniform random points within the plane described by the figure. Darker shades correspond to higher probabilities of persistence. Red symbols show features of the distribution for dog population sizes (estimated from the demographics data) and the posterior distribution of R_0_ in large shelters (estimated from seroprevalence data by MCMC; see Figures 2 and 3), including the median (hollow circle), mean (filled circle), 2.5th percentile (minus sign) and 97.5th percentile (plus sign). (**B**) Results of an intervention that reduces the arrival rate of susceptible individuals at a shelter to 1/<R_0_> its current value, where <R_0_> is the mean posterior distribution of R_0_ for CIV estimated from all available data. A kernel density estimate for the distribution of shelter sizes in the demographic data is shown below (**A**) and (**B**) to illustrate the scarcity of shelters large enough to support CIV in an endemic state.

Using the shelter demography data and the stochastic epidemic model, we simulated CIV outbreaks in shelters of a realistic distribution of sizes, intake rates and output rates, and for varying levels of R_0_. From these simulations we estimated the probability that a shelter (of a given population size, N) infected with a CIV virus (of a given R_0_) could maintain the virus for 100 days. The response surface for this experiment yielded a cut-off curve in the N-R_0_ plane, below which fadeout was almost certain and above which persistence was almost certain (Figure 4A, shaded surface).

Interestingly, the posterior distribution for R_0_ (estimated by MCMC as described above), and the empirical distribution of shelter population sizes from the demographic data, suggest that CIV straddles the border between persistence and stochastic fadeout. That is, the demographic and seroprevalence data indicate that CIV cannot persist in the majority of shelters, because the median of the posterior distribution of R_0_ is below the cutoff for persistence in a shelter of median size. But CIV can persist in existing shelters that are larger than the median size, because the posterior distribution for R_0_, combined with the distribution of shelter sizes from the demographic data, has significant density at R_0_-N combinations that would allow persistence (Figure 4A, points). This suggests that CIV persists on the brink of extinction—its current transmission efficiency is only sufficient to persist in large, high-throughput populations, but not yet to invade more widely.

More generally, for CIV in animal shelters the stochastic epidemic simulations parameterized with demographic data reveal that the impact of demographic stochasticity is considerable; the majority of shelters are too small to maintain the virus in the long term at its present rate of transmission. Thus variation in contact rates among host subpopulations, rather than inherent limitations on the evolved transmission efficiency of the pathogen, is sufficient to explain observed heterogeneity in prevalence in CIV, since the virus has been shown to transmit efficiently in large shelters and under laboratory settings [23].

### Control and Eradication Strategies

We then used the parameterized epidemic model and demographic data to simulate the impact of control strategies for CIV. The purpose of this analysis is to test how the observed hotspot dynamics are predicted to interact with potential eradication strategies, and to estimate, given available demographic and epidemiological data, the degree of control efficacy required to eliminate CIV from animal shelters where it is persisting, allowing for the complete eradication the virus from the canine population. Figure 4B shows the effect of a generic control measure applied to a single shelter, that reduces the inflow of susceptible dogs to a proportion 1/<R_0_> = 0.26 of current levels, where <R_0_> = 3.9 is the mean of the posterior distribution for R_0_, as above. This generic control measure has the effect of reducing the effective reproductive number in a single shelter to unity. Consistent with theory [38] this is a reduction in the susceptible portion sufficient to eradicate the disease from a large isolated shelter.

We then simulated a control program across multiple shelters connected by the transfer of dogs. The simulated control measure applied to the metapopulation is represented in the model as a vaccination program, but the results apply to any control that achieves the same reduction in the force of infection. A possible strategy (but to our knowledge untested; see below) that might be used is a live attenuated influenza vaccine (LAIV) that is able to generate an interfering response (possibly through generation of interferon) which prevents infection by wildtype CIV.

**Figure 5:**
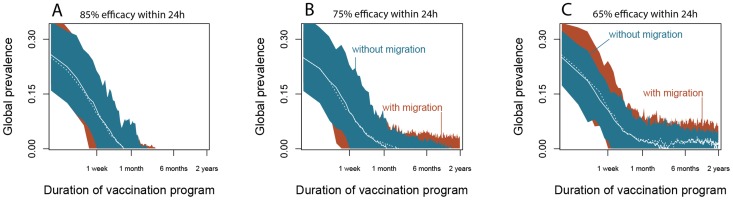
Predicted performance of a control program administered to dogs on arrival in US animal shelters. (**A**) A vaccination (or other control measure) that removes individuals from the chain of transmission with 85% probability (κ = 0.15) within 24 h (α = 1 day) is predicted to eradicate CIV from shelters within six months. The simulations used 100 shelters with dog population size, intake rate, and outtake rate jointly sampled with replacement from the shelter demographics data, and R_0_ = 3.9. White lines show medians and shaded areas enclose the 5^th^ to the 95^th^ percentiles of the simulation data. (**B**) Decreasing control efficacy to 75% can still achieve eradication in isolated shelters (blue region, solid line), however shelters that transfer dogs amongst themselves at the observed mean rate of τ = 0.1 would preserve CIV in a few shelters despite the vaccination program (red region, dashed line). (**C**) Further decreases in vaccine efficacy make eradication significantly less likely, particularly if shelters are connected through the transfer of dogs.

We find that such a control program could eradicate CIV within 1–2 months if it is applied to dogs immediately upon arrival to the shelter, and removes them from the chain of transmission within 24 hours with 85% probability (Figure 5A). A control strategy that has an efficacy of 75% might also efficiently eradicate CIV from isolated shelters, but transfers of dogs between shelters at the observed mean rate will allow CIV to persist through connected chains of outbreaks (Figure 5B). Control strategies with efficacies of 65% or less would reduce the prevalence of the infection, but are not predicted to lead to CIV eradication in every case (Figure 5C). In addition, without appealing to models it is clear from the demographic data that turnover rates in most shelters are too high for an inactivated vaccine to be effective, because those vaccines take more than a week to generate protective immunity. Since the expected residence time of a dog in an animal shelter is around 10 days, most dogs would have been part of the chain of transmission by the time an inactivated vaccine given at intake took effect, and then they would leave the shelter.

We therefore suggest one possible approach for control is the development of an LAIV administered at intake of dogs into shelters, which may provide suppression of the wild type virus infection by direct competition for the target tissues, as well as through stimulation of innate immune responses. This potential approach requires further research. In addition we note that the LAIV performance required to eradicate CIV is higher than what has been typically observed in other live influenza vaccines, and there is still much uncertainty about the rate at which protection would be acquired during the time that dogs spend in shelters (typically 1–2 weeks) [39]. However, other control measures (e.g. quarantine, decrease in population size, changes in population structure, or anti-viral drugs) or combinations that removed dogs from the chain of transmission with similar efficiency would also have qualitatively equivalent effects in a control strategy.

The key result is that a net control efficacy of ∼85% is required for eradication of CIV from its reservoir in a network larger animal shelters, even though a net efficacy of ∼75% would probably be sufficient for eradication from a single shelter. Thus another interesting feature of the control simulations in a metapopulation framework is the sensitivity to migration parameters (Figure 5). In particular, we show that there are efficacious control strategies that would cause the virus to go locally extinct but that would fail to achieve global eradication. These include scenarios where the expected global prevalence approaches 0, but where the virus would persists through connected chains of stochastic outbreaks (Figure 5B).

**Figure 6:**
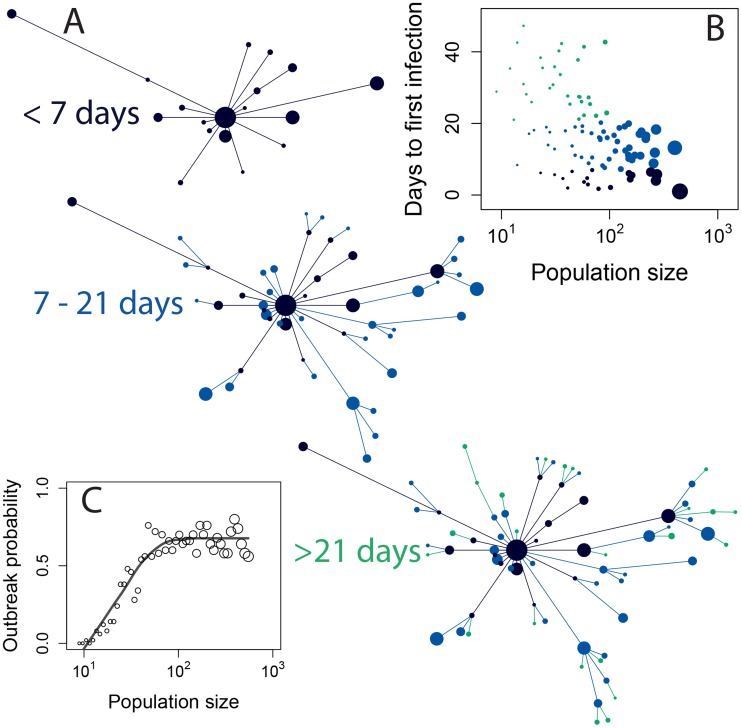
A simulation of CIV invasion over multiple shelters, starting with an infection in a single large shelter. (**A**) Each vertex represents an animal shelter with dog population size proportional to the area of the circle. Edges show transfer of infection from shelter to shelter over time through the movement of infected dogs. Edge lengths are arbitrary. The data for this figure were produced by simulating the metapopulation stochastic SIR model with 100 shelters for 100 days, starting with a single infection in the largest shelter. Population sizes were sampled with replacement from the shelter data. R_0_ = 3.9. Transfer probability is set to the mean observed value of τ = 0.1. (**B**) Large shelters tend to receive the infection earlier (and more often) following an outbreak at another shelter. (**C**) Probability that CIV will persist for 100 days in a shelter of a given size following the introduction of a single infected individual to an otherwise susceptible population. The plateau on the curve arises for populations sufficiently large that early depletion of susceptibles is not an important factor in the probability of an outbreak: rather than population size, this probability is determined by R_0_.

We also used our epidemic model to explore the passage of CIV from an infection in one large shelter to other shelters through the transfer of dogs (Figure 6A). The hotspot dynamics predicted by our model show regularities in the way CIV spreads outward from a single shelter. Large shelters are predicted to receive the infection earlier, as well as maintaining it for longer, creating a wave in the population size—time-of infection plane (Figure 6B). The probability that a single infection introduced to a susceptible shelter would start an epidemic that persisted for at least 100 days increases with population size (Figure 6C). For the median population size of 43 dogs the probability was approximately 0.5.

## Discussion

Since its emergence more than a decade ago, equine H3N8-derived CIV has maintained a patchy distribution, occurring most often in sporadic and short-lived outbreaks in US animal shelters [21]. In contrast, strains of CIV's recent ancestor (EIV H3N8) have been commonly found in horses around much of the world since at least 1963. EIV thus transmits efficiently among horses, sometimes despite vaccination programs [33], [40]. Our study investigates heterogeneity in CIV transmission and prevalence to better understand the processes that determine how zoonotic pathogens spread following a host range shift, and how these processes affect the performance of control and eradication strategies.

Stuttering chains of infection in recently emerged zoonoses are caused by two distinct mechanisms that may operate in tandem. The first is poor adaptation of the pathogen to its new host species, causing lower within-host replication rates that may reduce pathogen shedding and lead to inefficient transmission [11]. Transmission is inefficient because many potentially infectious contacts between infected and susceptible individuals fail to spread the infection. The second mechanism that generates stuttering chains is variation in contact rates among different subsets of the new host population, which can increase the probability of stochastic fadeouts [13]. In this case the fadeouts are not caused by inefficient transmission but by exhausting the local supply of susceptible hosts.

Strikingly, host contact heterogeneity has received less attention as a potential driver of stuttering chains despite its fundamental role in disease dynamics. We found that contact heterogeneity plays a critical role in the patchy distribution of CIV. Demographic data indicate that most shelters are too small, and import susceptible individuals too slowly, to protect CIV from local stochastic extinction at its current reproductive rate. At the same time, the influence of contact heterogeneity on the spread of CIV would diminish if transmissibility were higher and the epidemiology of CIV (and similar zoonoses) generally represents an interaction between contact heterogeneity and transmission efficiency.

More evidence of contact heterogeneity appears at the inter-shelter scale. Observed transfer rates suggest that the majority of intakes and outputs are not associated with other shelters, so that shelter-to-shelter transfer has not created an effectively larger metapopulation of multiple shelters. While there are many millions of susceptible household dogs in the USA (around 80 million, about 25% the size of the human population), it is likely that they do not exhibit infectious contact patterns sufficient to maintain the virus in continuous transmission (see below). Hence, this work shows that apparent stuttering chains of transmission are not always driven by poor adaptation of an emerging pathogen to its new host. Rather, in the case of CIV, demographic variability in contact rates is alone sufficient to explain the fadeout in the disease in most situations.

The basic reproductive number for CIV is intriguingly close to the minimum value required to persist in a shelter of average size (Figure 4A). This suggests that transmission efficiency in CIV may have evolved to precisely the point of persistence. Yet, this average shelter size belies the high variance in shelter populations, with many small facilities balanced by a few extraordinarily large ones (roughly matching the distribution pattern of US city sizes; Figure 2A). The process by which CIV currently persists is therefore to thrive in a few large populations with high rates of infected-susceptible contact (as mediated by a high arrival rate of new susceptibles, rather than by increase density), but failing to take hold in the general population (Figure 4A). By analogy with conservation biology, large populations thus function as refugia for the virus, protecting it from extinction and also limiting its distribution. These are also exactly the conditions to facilitate further evolution toward higher reproductive capacities, by facilitating repeated outbreaks outside the refugia, followed by selection for higher transmissibility [10], [12], [41].

Our mean estimate of R_0_ = 3.9 in the large animal shelters is lower than that estimated for EIV during outbreaks [42], but close to the upper bound for estimates of human influenza transmission [43], [44]. It is also considerably higher than that of pandemic H1N1 influenza in humans in 2009 (R_0_ = 1.4–1.6) which spread worldwide within weeks of its first recognition in humans [45]. Variation in R_0_ among different viral strains and host species can be difficult to interpret because of the many factors that can affect transmission and removal rates in different settings. However, these comparisons do indicate that CIV has the biological capacity to spread relatively efficiently among dogs given the right conditions in the host population.

Although most of the parameters in the epidemic model were estimated from a large volume of host demographic data from animal shelters, there are currently few estimates of seroprevalence in shelters where CIV is endemic and as a result our estimate for equilibrium seroprevalence relies on data from a single shelter [25]. This introduces a risk of sampling bias because that individual shelter could exhibit individual characteristics that affect its equilibrium seroprevalence, and/or due to non-random temporal fluctuations in seroprevalence. While the scarcity of seroprevalence estimates adds uncertainty to R_0_ estimates, the extant data would be difficult to explain with values of R_0_ lower than our estimates. This is due to the rapid rate at which infected individuals are replaced by new arriving susceptibles in the high throughput shelters where CIV is enzootic. The low residence time of dogs in large, high-throughput shelters thus indicates (consistent with previous results [25]) that individuals in shelters where CIV is enzootic must acquire the infection within a few days of arriving. This places a lower bound on probable values for R_0_ by constraining estimates of the generation time of the infection, at least in the context of large shelters [44]. In other facilities that are smaller or where CIV is not enzootic, long-term average seroprevalence over time may be lower [26] due to stochastic fadeouts of the disease.

Phylogenetic analyses independently supports several key predictions of our analysis. First, these analyses confirm that CIV remains confined to endemic hotspots, with transfers to other regions causing outbreaks that are generally short-lived (and thus failing to establish new lineages outside of endemic locations). Moreover, the strong phylogeographic structure, with distinct viral clusters in New York, Pennsylvania and Colorado for each gene analyzed, is exactly what might be expected given our empirically-parameterized epidemic model, which predicts geographic segregation. Each US state has only a few large cities that would have an animal shelter capable of supporting CIV in the long term, and with relatively infrequent transfers among cities.

Second, the mean and HPD interval for R_e_ from the phylogenetic analysis in 2004 (when CIV was first detected and when infected greyhounds were being transported to many US states for racing) roughly matches the distribution of R_0_ from the stochastic SIR model and demographic data. The initial phase of an epidemic is usually associated with exponential growth (corresponding to R_e_ = R_0_), and R_e_ must always be less than or equal to R_0_ by definition. This makes the phylogenetic estimate of R_e_ in 2004 a conservative independent assessment of R_0_.

The third concurrence between the demographic and phylogenetic analysis involves the current estimate of R_e_∼ = 1. While R_0_ in our analysis measures the reproductive potential of the disease where it is enzootic, the phylodynamic estimates of R_e_ reflects the net spread rate of CIV across the US as a whole, including multiple shelters and the companion dog population. As such, R_e_∼ = 1 indicates that on balance CIV is currently failing to persist where it is not already enzootic, which is consistent with both epidemiological observations [21] and the predictions of the demographic analysis which showed that most shelters are too small to support CIV in an endemic state (see Figure 4).

Targeted strategies for control and eradication depend on understanding the conditions under which an emerging enzootic pathogen can maintain itself in a new host population. Our simulations indicate that eradication may be possible, but will require relatively efficient control, and that success may depend on the rates at which dogs are transferred between animal shelters. For control measures with ∼75% efficiency, CIV may be eradicated from most shelters but still persist overall through connected chains of outbreaks, with large shelters serving as focal points for staging new infections elsewhere. This suggests that control programs for CIV will be most successful if implemented across multiple shelters and that participating shelters should maintain control measures even after the virus has been eliminated locally.

Uncertainties in our analysis would be reduced by more information on CIV prevalence and additional CIV sequence data. A key area of uncertainty is transmission rates between companion dogs within and among households whose contact patterns would differ significantly from those of dogs in animal shelters. Working from first principles, if a proportion *p* of contacts between infectious and susceptible dogs result in the infection being transmitted, then the probability that an infected individual in the companion population will first transmit the infection on their *k*th contact with a susceptible dog is *p(1-p)^k−1^*, which gives an expected value for *k* of *1/p*. Studies of CIV transmission in comingling trials estimate *p = 0.75* [23]. This suggests that for a CIV lineage to avoid extinction in companion dogs, the average infected dog must contact k = 1.33 susceptible individuals during the time they are infected. Another approach that yields the same result is to recognize that if a proportion *p* of contacts produce secondary infections, then R_0_>1 requires *k>1/p*, again translating to approximately two contacts per week. It is evident from common experience that while some companion dogs are highly sociable, others do not frequently interact with other dogs. Therefore, some companion dogs probably achieve the minimum contact rate required to sustain CIV, but others probably do not. Many dogs with higher contact rates than this minimum would be necessary for CIV to actively spread among companion dogs, and to protect the virus from stochastic extinction in the general dog population, at its current transmission efficiency.

Conversely, if CIV had emerged with much higher transmissibility upon entry to the greyhound population, stuttering chains of transmission would not have been observed. Thus, the capacity for contact heterogeneity to control epidemic spread is facilitated by lower transmissibility. Furthermore, while endemic hotspots created by contact heterogeneity can theoretically increase the chances of evolving higher transmission efficiency, it is evident that not all cross-species transmission events will involve small stepwise gains in transmissibility in the new host. Indeed, a variety of adaptive models, involving differing numbers and fitness of mutations, can be put forward to explain the process of emergence [46].

Despite these caveats, and limitations in the available data, our analyses provide a coherent view of the ecological and evolutionary dynamics of CIV. After approximately 15 years of continuous circulation among dogs in the US, CIV can be maintained only in relatively dense host populations with high inputs of susceptible individuals (essentially viral “chemostats”), despite a relatively high reproductive potential in that context. These hotspots are weakly connected by migration, leading to geographic signatures in the CIV phylogenies. Most dog populations are too small or diffuse to independently support CIV at its current level of transmissibility, explaining its current modest reproductive rate (R_e_∼ = 1), and consistent with the epidemiology of CIV, which is characterized primarily by sporadic short-lived outbreaks outside of enzootic centers [21]. The demographic gradient between high-throughput populations where CIV is enzootic, and smaller or more diffuse populations where sporadic outbreaks can occur, creates hotspot dynamics that can facilitate pathogen evolution toward higher transmissibility [12], [41]. Our results therefore demonstrate one way that urbanization can increase the risk posed by emerging infectious pathogens [2], [47].

Although humans exposed to CIV appear not to be commonly infected (as shown by serological testing), the true risk of future human infection by either EIV or CIV is unknown as we do not understand the host barriers that restrict human infection, or the genotypic changes in the viruses that might overcome those barriers [19]. Our analysis can therefor inform a strategy for preemptive eradication of an influenza A virus that is well adapted to mammals, since if CIV did gain high transmissibility among companion dogs then much of the human population would be directly exposed to the virus.

## Methods

### Epidemiological Model

Our analysis is based on an SIR framework that models changes over time in the number of dogs in a shelter who are susceptible (S), infected (I), or removed (recovered and thus immune; R). In what follows, we describe the model for a single shelter: below we expand the model to incorporate the dynamics of an control program that reduces the force of infection, and to consider control in multiple shelters linked through the transfer of dogs.

We assume dogs arrive at a shelter of a given size at a rate of μ dogs per day. Dogs leave at a per-capita rate of δ per dog per day, regardless of their state, so the mean residence time in a shelter is 1/δ days. The number of dogs in a shelter, N = S+I+R, is equal to μ/δ at equilibrium. Arrival and departure rates are estimated empirically using individual-level records from 13 animal shelters of varying size across the US (see Supporting Information, Data S1). The records comprise a total of 124,519 dogs, recording the date each individual arrived and left the shelter. In 8 of the 13 shelters, the data included whether or not the departure of the dog represented a transfer to another shelter. Arrival rate, μ, for a shelter was estimated as the median number of dogs arriving in that shelter per day. Departure rate, δ, for a shelter was estimated as the inverse of the median length of stay of dogs in that shelter. When estimating arrival and departure rates we excluded dogs that were admitted to the shelter in response to a euthanasia request, as these dogs had systematically shorter residence times. We also excluded dogs whose length of stay was greater than 40 days, as these represented rare atypical cases (see Figure 2C,D).

We assume that dogs in a shelter have a constant rate of contact per day with other dogs where the contact would be capable of spreading infection if one of the dogs were infected. An alternate hypothesis is that contact rate increases with population size, potentially leading to hotspot dynamics in large shelters in the absence of demographic stochasticity. Our assumption of constant contact rates is thus conservative with respect to the hypothesis that demographic structure drives hotspot dynamics in CIV.

Assuming that contact between any pair of dogs in the shelter is equally likely, the force of infection is given by λ = βP, where β is the contact rate and P = I/N is the current prevalence of CIV in the shelter [48]. The rate of new infections is given by λS, and susceptible dogs contract the disease an average of 1/λ days after entering the shelter. In this framework, the basic reproductive number of the disease is R_0_ = β/(γ+δ), and the disease only persists in the long run if R_0_>1, in which case equilibrium prevalence is given by
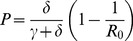
(1)which is bounded above by δ/(γ+δ) as R_0_ becomes large (see Supporting Information, Text S1, Section 1.6).

The infected class in our model represents the number of dogs with non-zero viral loads, rather than those exhibiting clinical symptoms. Thus, we avoid including latent or asymptomatic classes in our model. We set γ = 1/7 because viral shedding continues for approximately seven days after inoculation [16]. Seroconversion for dogs infected with CIV also happens at approximately 7 days [16]. Equilibrium seroprevalence is then given by R/N (see Supporting Information, Text S1, Section 1.7).

Variation among individuals in time of infection, recovery, arrival, and departure causes variations in disease prevalence around the predicted long-term average. These excursions from mean prevalence carry with them the risk of visiting zero prevalence, leading to stochastic extinction of the disease. This demographic stochasticity becomes increasingly pronounced in smaller populations. However, the critical population size below which disease dynamics begin to significantly diverge from the long-term average through stochastic fadeouts depends upon R_0_, and upon the turnover rate in the population. We parameterize the stochastic SIR model with the demographic data to test the impact of demographic stochasticity on the spread and maintenance of CIV in animal shelters. We implement the model in continuous time at the level of individual dogs using the Gillespie algorithm [37].

We estimated a posterior distribution for R_0_ given seroprevalence data and demographic data by using a Markov Chain Monte Carlo (MCMC) method, as follows. From the stochastic SIR model we simulated seroprevalence samples by observing the seropositivity of *n* randomly selected dogs from the population at a given time. Seroprevalence thus observed has the property of being normally-distributed about the long-term equilibrium value given by the mean-field model in our simulations (Figure 3B). We then seek the posterior distribution of an unknown equilibrium seroprevalence at an actual shelter, given a real point seroprevalence estimate there. We estimate this distribution by sampling from the Gaussian distribution of deviations between point seroprevalence estimates and equilibrium seropreovalence, using the Metropolis-Hastings algorithm [49]. We assessed convergence by visually examining within- and among- chain mixing. Convergence was determined to have occurred when the long-term variance in sampler state among chains was the same as the variance within chains. Convergence under this definition was easily achieved using 10 chains run for 10^5^ steps each, with a burnin of 10%, and keeping every 100^th^ step. From the posterior distribution of equilibrium seroprevalence, we then map to a posterior distribution for R_0_ by inverting Equation 1.

### Control and Eradication Strategies

We represent control strategies by a vaccination program that reduces the force of infection (as described below), but the results apply to any control measure that provides a similar reduction in risk of infection and in infectiousness. In what follows we discuss control in the context of a LAIV administered to dogs upon arrival at the shelter.

The model with vaccination dynamics includes two more compartments, counting the number of dogs in each shelter who are vaccinated (V), and the number of dogs who are infected despite vaccination (W). Vaccination reduces a dog's susceptibility to infection by decreasing the probability that a virus population initially transferred through infectious contact will enter a phase of exponential growth, prerequisite to significant viral shedding and clinical symptoms [50].

By reducing viral load and viral shedding, vaccination reduces the risk of infection in vaccinated dogs and reduces the infectiousness of a dog who becomes infected despite vaccination. Vaccinated dogs thus experience a reduced force of infection ελ, 0≤ε≤1, and, if they become infected, contribute to the force of infection at a reduced rate 0≤ω≤1, leading to an overall force of infection of λ = β(I+ωW)/N in population which has W vaccinated individuals who have nonetheless become infected.

Dogs transition from S to V at a rate of α per dog per day. Because a live vaccine is assumed to be administered to dogs immediately upon arrival, 1/α measures the average time after entry/vaccination that a dog experiences the vaccine-associated decrease in risk of infection from other dogs, and decreased infectiousness if they do become infected. Vaccination changes mean dynamics by reducing R_0_ by a factor of 1-K, where K is effective vaccination coverage. K is given by (1-κ)V/N, where κ = εω expresses the failure rate of the vaccine, ranging from 0 for perfect vaccine, to 1 for an entirely ineffective one (see Supplementary material, section 1.4). We use a step function for κ as a function of α, where κ goes from 1 to its post-vaccine value at 1/α days.

We also model the effects of a generic control strategy equivalent to inoculating some dogs with a perfect vaccine, or to quarantine that partially or completely stops the flow of susceptible dogs into the shelter. We do this by replacing susceptible dogs with removed ones in the intake stream. Reducing the proportion of susceptible dogs in the intake stream to 0<*θ*<1, while 1- *θ* are already removed, has the same effect as reducing R_0_ to *θ*R_0_.

### Metapopulation Dynamics

The metapopulation model expands the stochastic SIR model for a single shelter to describe multiple shelters whose dynamics are linked by the transfer of dogs. As above, the model is implemented at the level individual dogs using the Gillespie algorithm. Thus at each point in continuous time, each individual in the model has a disease state (S,I,R,V, or W) and a location in a given shelter. The metapopulation is composed of shelters that vary in dog population size, intake rate and output rate by sampling with replacement from the demographic data. Transfer probabilities are also based on the demographic data (see Supporting Information, Text S1, Section 2). Although the CIV phylogenies show geographic localization (see Figure 1), the metapopulation model is spatially implicit, consistent with level of detail in the demographic data we used. However, even without including spatial structure in transfer patterns, the metapopulation model reproduces hotspot dynamics, based on transfer hierarchies driven by differences in shelter size (see Figure 6).

### Phylogenetic Analysis, Estimates of R_e_, and Phylogeography

We compiled all available CIV HA1, NP and M gene segment sequences from GenBank and the Influenza Research Database (www.fludb.org) and by sequencing samples provided by the Animal Health Diagnostic Center (AHDC) at Cornell University. For the sequencing of the virus samples obtained from AHDC we extracted viral RNA using Qiagen viral RNA mini kit and synthesized cDNA using Avian Myeloblastosis Virus (AMV) reverse transcriptase and influenza universal primer Uni12. Three gene segments, HA1, NP and M, were then amplified by PCR with gene specific primers (primer sequences are available upon request) for all samples. The PCR products were purified using EZNA Cycle-Pure Kit and sequenced by the Sanger method. All sequences derived here have been submitted to GenBank and assigned accession numbers KM359803-KM359864.

All sequences were aligned by MUSCLE v3.8.31 [51] using default parameters, followed by manual adjustment. Phylogenetic trees of each gene were then estimated using the maximum likelihood (ML) available in PhyML 3.0 [52] and assuming the general time-reversible reversible (GTR) model of nucleotide substitution and a gamma distribution of among-site rate variation with 4 rate categories (i.e. the GTR+I+Γ_4_ model of nucleotide substitution) with SPR branch-swapping. The robustness of the phylogeny was estimated using 1,000 bootstrap replicates. Because of their greater availability, the analyses of evolutionary dynamics and phylogeography were only performed on the HA1 gene (see below).

To estimate R from the CIV sequence data we used a total of 94 HA1 sequences (alignment length = 1032 nt) sampled from various locations (states) in the US (Colorado, New York, Pennsylvania, Florida, California, Kentucky, Wyoming, Philadelphia, South Carolina, Virginia, Vermont, Connecticut, Texas, and Iowa) between 2003 and 2013. This data set included 40 sequences sampled from dog shelters in New York between 2005 and 2012, which were analyzed separately using the same protocols.

First, we estimated the mean (and credible intervals) of R in both data sets using the epidemiological birth-death method [53] available in BEAST v1.7.5 [54]. This analysis used the simpler Hasegawa-Kishino-Yano (HKY) model of nucleotide substitution and a gamma distribution of among-site rate variation (HKY+Γ_4_). To account for temporal rate variation in the data an uncorrelated lognormal relaxed molecular clock model was employed. Using the Bayesian Markov Chain Monte Carlo (MCMC) framework available in BEAST, 100 million steps were run, sampling every 10,000 and removing 10% as a burn-in. Second, temporal changes in R were estimated using the more complex serial-sampled birth-death (SSBD) model [15], available in BEAST v2.0 [55], again using the HKY+Γ_4_ but this time (to ensure statistical convergence) employing a strict molecular clock with a uniform distributed clock rate of 2×10^−3^ (1×10^−3^–3×10^−3^) nucleotide substitutions per site, as this was found to be best-fit to the data in epidemiological birth-death method. The MCMC was again run for 100 million steps, sampling in the same way as described above. Two independent runs allowed different R_e_ values to be inferred from up to R_e_ = 25.

To determine whether CIV was more clustered on the phylogenetic tree by US state of sampling than expected by chance alone, we employed the Association Index (AI), Parsimony Score (PS) and Maximum Clade size (MC) phylogeny-trait association statistics incorporated within the Bayesian Tip-association Significance testing (BaTS) program [36]. Traits were defined as the US state of sampling. Phylogenetic uncertainty in the data was incorporated by basing estimates on the posterior distribution of trees obtained from the BEAST analysis (epidemiological birth-death method) described above. In all cases, 1000 random permutations of sampling locations were undertaken to create a null distribution for each statistic.

## Supporting Information

Data S1 **State-of-origin of the animal shelters.**
(XLSX)

Text S1 **Details of the epidemic model.**
(PDF)
