# Supplementary material for: Contact Heterogeneity, Rather Than Transmission Efficiency, Limits the Emergence and Spread of Canine Influenza Virus
Source: PLoS Pathog. 2014 Oct 23;10(10):e1004455. doi: 10.1371/journal.ppat.1004455 (PMC4207809; doi:10.1371/journal.ppat.1004455)
Supplement: Text S1 — Details of the epidemic model. (PDF) [file ppat.1004455.s002.pdf]

Supporting Information for Dalziel et al. 2014. Contact Heterogeneity, Rather Than Transmission Efficiency, Limits the Emergence and Spread of Canine Influenza Virus

1 Details of the epidemic model for CIV in animal shelters

1.1 Mean field, single shelter

$$\dot{S} = \mu - (\lambda + \alpha + \delta)S \quad (1)$$
$$\dot{I} = \lambda S - (\gamma + \delta)I \quad (2)$$
$$\dot{R} = \gamma(I + W) - \delta R \quad (3)$$
$$\dot{V} = \alpha S - (\varepsilon\lambda + \delta)V \quad (4)$$
$$\dot{W} = \varepsilon\lambda V - (\gamma + \delta)W \quad (5)$$

where

$$\lambda = \frac{\beta(I + \omega W)}{N} \quad (6)$$

and the "dot notation" on the left hand side is short for the time derivative of a state variable, e.g. $\dot{X} \equiv dX/dt$.

The base model without vaccination is a special case where $\alpha = 0$, and $V = W = 0$

1.2 Disease-free equilibrium

$$S = \frac{\mu}{\alpha + \delta} \quad (7)$$
$$V = \frac{\alpha}{\alpha + \delta}N \quad (8)$$
$$(9)$$

where $N = \mu/\delta$.

1.3 R_0 from mean field absent vaccination

We use the spectral radius method [1]. Let x represent the state vector for the system, so that the ith element of x corresponds to the value of the ith state variable. Let x_0 represent the disease free state. Let $\mathcal{F}_i(x)$ be the rate of appearance of new infected individuals into class i and $\mathcal{V}_i(x)$ represent the rate at which infected individuals leave that class. Define the matrices $\mathbf{F} = [\mathbf{F}_{ij}]$ and $\mathbf{V} = [\mathbf{V}_{ij}]$ such that

$$\mathbf{F}_{ij} = \frac{\partial \mathcal{F}_i}{\partial x_j}(x_0) \tag{10}$$

and

$$\mathbf{V}_{ij} = \frac{\partial \mathcal{F}_i}{\partial x_j}(x_0) \tag{11}$$

where i and j cover only the classes of infected individuals. Then

$$R_0 = \rho\left(\mathbf{F}\mathbf{V}^{-1}\right) \tag{12}$$

where ρ is the spectral radius of the resulting matrix.

Absent vaccination, we have

$$\mathcal{F} = \frac{\beta}{N} SI \tag{13}$$

and

$$\mathcal{V} = (\gamma + \delta) I \tag{14}$$

This leads to

$$R_0 = \frac{\beta}{\gamma + \delta} \tag{15}$$

1.4 R_0 from mean field, full model

Vaccine coverage, C, at the disease free equilibrium is

$$C \equiv \frac{V}{N} = 1 - \frac{S}{N} = \frac{\alpha}{\alpha + \delta} \tag{16}$$

Then

$$\mathbf{F} = \beta \begin{bmatrix} 1 - C & \omega(1 - C) \\ \varepsilon C & \varepsilon \omega C \end{bmatrix} \tag{17}$$

and

$$\mathbf{V} = (\gamma + \delta) \begin{bmatrix} 1 & 0 \\ 0 & 1 \end{bmatrix} \tag{18}$$

so

$$\mathbf{F}\mathbf{V}^{-1} = \frac{\beta}{\gamma + \delta} \begin{bmatrix} 1 - C & \omega(1 - C) \\ \varepsilon C & \varepsilon \omega C \end{bmatrix} \tag{19}$$

Letting R_0^+ represent the R_0 under vaccination, we have

$$R_0^+ = \rho\left(\mathbf{F}\mathbf{V}^{-1}\right) \tag{20}$$

$$= \frac{\beta}{\gamma+\delta}\left(1+\varepsilon\omega C - C\right) \tag{21}$$

This can be made more familiar by substituting the vaccine-free R_0 in place of $\beta/(\gamma+\delta)$ and defining K as the effective coverage, adjusted for vaccine performance,

$$K \equiv (1-\varepsilon\omega)C \tag{22}$$

to yield

$$R_0^+ = R_0\left(1-K\right) \tag{23}$$

1.5 Endemic equilibrium absent vaccination

Starting with the I-nullcline

$$\dot{I} = 0 \tag{24}$$

$$\iff S = \frac{\gamma+\delta}{\beta}N \tag{25}$$

$$\iff \frac{S}{N} = \frac{1}{R_0} \tag{26}$$

Then the S-nullcline

$$\dot{S} = 0 \tag{27}$$

$$\iff I = \frac{\mu N - \delta S N}{\beta S} \tag{28}$$

$$= \frac{\mu}{\beta}\frac{N}{S} - \frac{\delta}{\beta}N \tag{29}$$

Substituting in $N/S = R_0$, the S-nullcline satisfies

$$I = \frac{\mu}{\beta}R_0 - \frac{\delta}{\beta}N \tag{30}$$

1.6 Prevalence

Prevalence at endemic equilibrium is given by

$$P \equiv \frac{I}{N} \tag{31}$$
$$= \frac{\delta}{\beta}(R_0 - 1) \tag{32}$$
$$= \frac{\delta}{\gamma + \delta} - \frac{\delta}{\beta} \tag{33}$$
$$= \frac{\delta}{\gamma + \delta}\left(1 - \frac{1}{R_0}\right) \tag{34}$$

1.7 Seroprevalence

Time to seroconversion is close to duration of viral shedding at around 7 days, so we assume recovered individuals have seroconverted and infected individuals have not [2]. Equilibrium (long-term average) seroprevalence in a shelter where CIV is endemic is thus given by

$$Z^* \equiv \frac{R}{N} \tag{35}$$
$$= 1 - P - S/N \tag{36}$$
$$= 1 - \frac{\delta}{\gamma + \delta}\left(1 - \frac{1}{R_0}\right) - \frac{1}{R_0} \tag{37}$$

2 Metapopulation model

We have individual level data for 124,519 dogs from 13 animal shelters, spanning 2008 to 2013. Each row in the resulting data frame consists of the arrival and departure dates of one dog from a specified shelter. The data also contained a column giving information on outcome type, allowing us to exclude dogs that were admitted as the result of euthanasia requests, as their residence times were systematically lower than that over other dogs. In the ith shelter we estimate the arrival rate μ_i as the median number of dogs arriving per day to that shelter over the duration of the observation period. The departure rate in shelter i is estimated by taking the inverse of the median length of stay for dogs in that shelter over the duration of the observation period. Equilibrium shelter population size for shelter i is then given as $N_i = \mu_i/\delta_i$. We reconstructed the shelter's actual population size over time by subtracting cumulative departures from cumulative arrivals, and visually checked the stationarity of each shelter's actual population size against the equilibrium value given by N_i. Eight shelters recorded whether outcomes were transfers to other shelters. In each of these eight shelters we calculated the proportion of all outcomes that were transfers. The average of this value across shelters is $\tau = 0.1$ (0.1 s.d). When estimating intake, output, and transfer we excluded dogs whose length of stay was greater than 40 days, as these represented rare atypical cases.

To parameterize the metapopulation model we need the arrival rate of dogs to each shelter from each other shelter μ_{ij}. We also need the per-capita rate at which shelter i transfers dogs to shelter

j, δ_{ij}. Let μ_{ii} represent arrivals from outside the metapopulation and let δ_{ii} represent the per-capita rate of outcomes that are not transfers to another shelter (e.g. adoption, return to owner, euthanasia).

Here is how we go from the demographic data we have to the metapopulation parameters we need. The mean per capita departure rate for the shelter associated with a randomly chosen dog (which we have) satisfies

$$\delta_i = \sum_{j=1}^{M} \delta_{ij} \tag{38}$$

where there are M shelters in the metapopulation.

A proportion τ of these outcomes are transfers to other shelters, and we have an estimate of τ. Assume that shelters accept dogs transferred from other shelters in the metapopulation proportional to the size of the recipient shelter, so that large shelters accept more transfers than small ones. For shelter i, define the proportion of the metapopulation outside of shelter i that resides in shelter j as

$$p_{ij} = \frac{N_j}{\sum_{k \neq i} N_k}, j \neq i \tag{39}$$

which we can also calculate from the demographic data we have.

Assuming the metapopulation is closed, so that no dogs are transferred to shelters not in the metapopulation, we get δ_{ij} as

$$\delta_{ij} = \tau \delta_i p_{ij}, j \neq i \tag{40}$$

Substituting into equation 38 yields,

$$\delta_{ii} = \left(1 - \tau \sum_{j=1}^{M} p_{ij} \right) \delta_i \tag{41}$$

Now let μ_{ij} represent the number of dogs that arrive to shelter i from j per day. Then

$$\mu_{ij} = \delta_{ji} N_j \tag{42}$$

We assume that during transfers dogs are selected at random from the shelter without regard to their disease state. Thus the probability that a susceptible dog is transferred is S_i/N_i and so on for the other classes.

Finally, we choose μ_{ii} to balance the relation

$$\mu_i = \sum_j \mu_{ij} \tag{43}$$

where the left-hand side is given by data.

In contrast to μ_{ij}, the flow represented by μ_{ii} is assumed to consist of entirely susceptible dogs, because the prevalence of CIV among dogs not in animal shelters is very low.

Our metapopulation approach ignores shelter proximity when calculating transfer probabilities, because we have no data on how transfer rates vary with geographic distance. We therefor use the mean transfer rate τ as an appropriately simple first approximation for transfer dynamics among shelters. The relative low mean transfer probability of $\tau = 0.1$ suggests that transfer of dogs among shelters may be infrequent enough to prevent panmixia. That is, on a continuum from completely isolated to completely intermixed, dog populations in animal shelters in the US are closer to being solitary than to being totally connected. This is broadly consistent with finding of geographic segregation in the phylogenetic data.

References

[1] P. van den Driessche, J. Watmough, *Math Biosci* **180**, 29 (2002).

[2] F. F. Jirjis, *et al.*, *Vet. Microbiol.* **144**, 303 (2010).
